# A multivariable model of ultrasound and biochemical parameters for predicting high-volume lymph node metastases of papillary thyroid carcinoma with Hashimoto’s thyroiditis

**DOI:** 10.3389/fendo.2024.1501142

**Published:** 2025-01-10

**Authors:** Xiao-hui Liu, Hong-qing Yin, Hong Shen, Xi-Ya Wang, Zheng Zhang, Xiao-feng Yuan, Qi Tang, Jun Shao

**Affiliations:** ^1^ Department of Ultrasound Diagnosis, Affiliated Kunshan Hospital of Jiangsu University, Kunshan, China; ^2^ Department of Medical Ultrasound, Affiliated Hospital of Jiangsu University, Zhenjiang, China

**Keywords:** high-volume lymph node metastasis, papillary thyroid cancer, Hashimoto’s thyroiditis, histopathology, ultrasonographic features

## Abstract

**Objectives:**

This study aims to develop a nomogram to predict high-volume (> 5) lymph node metastases (HVLNM) in papillary thyroid carcinoma concomitant with Hashimoto’s thyroiditis by combining ultrasound with clinicopathologic data.

**Materials and methods:**

The study reviewed 187 patients diagnosed with papillary thyroid cancer (PTC) concomitant with Hashimoto’s thyroiditis from the First People’s Hospital of Kunshan between March 2018 and December 2022. These patients underwent preoperative ultrasound and postoperative examinations. They were divided into two groups based on the size of their lymph nodes (LNs). A predictive model was developed using LASSO regression and multifactor logistic regression analysis. The receiver operating characteristic (ROC) curve was used to validate the predictive model.

**Results:**

A total of 187 patients were randomized into 132 participants for training and 55 participants for external validation. Four predictors including tumor size, extrathyroidal extension, histological grade and vascularity, were selected from 13 variables based on LASSO regression analysis. In the training set, the model built from the above four predictor has a satisfactory predictive power, with an area under the ROC curve of 0.914, and validation set with the ROC curve of 0.889, which indicated that the nomogram can be used effectively in clinical settings.

**Conclusion:**

In summary, the nomogram constructed by tumor size, extrathyroidal extension, histological grade and vascularity, is useful for predicting the risk of HVLNMs in patients with papillary thyroid carcinoma associated with Hashimoto’s thyroiditis, which is expected to provide the basis for adequate and accurate management before the primary surgery.

## 1 Introduction

Thyroid cancer (TC) is the most common endocrine malignancy worldwide, with the increasing incidence over the past three decades (1, 2). Papillary thyroid carcinoma (PTC) represents the predominant subtype of TC, accounting for approximately 70% to 85.9% of all diagnosed cases (3). It is well-known that Hashimoto’s thyroiditis (HT) is an autoimmune disorder predominantly characterized by lymphocytic infiltration interspersed with epithelial (follicular) cells within the thyroid parenchyma (4, 5). Epidemiological studies report an average coexistence rate of 23% (ranging from 5% to 85%) between HT and PTC (6, 7), and patients diagnosed with PTC concomitant with HT exhibit a higher propensity for developing lymph node metastases (LNM) (8). Large-volume or high-volume lymph node metastasis (HVLNM) was characterized by the presence of more than five metastatic lymph nodes (9). Several studies indicate that patients with HVLNM exhibit poorer prognoses than those with small-volume LNM, characterized by elevated recurrence rates and reduced disease-free survival (10, 11). However, the desirability of prophylactic central lymph node dissection (CLND) in patients with enlarged cervical lymph nodes at initial screening is rarely discussed, especially given the increased risk of surgical completion and postoperative complications (12). In addition, there is few publication addressing the clinical necessity of prophylactic LND in PTC patients with significant lymph node enlargement in the cervical region.

Ultrasound (US) is the preferred modality for routine thyroid examinations and preoperative staging of thyroid cancer. Ultrasound examination is more accurate when there is localized liquefaction or internal punctate echogenicity in metastatic lymph nodes (13–15). Despite this, metastatic and inflammatory hypertrophic lymph nodes share common characteristics, including roundness and lack of fat gates (12, 13). Additionally, the complex anatomy of the central region of the neck reduces ultrasound’s sensitivity in identifying CLNM (14). Ultrasound has limitations in detecting central lymph node metastases in PTC with HT due to the proliferative inflammatory responses in the lymph nodes. The nature of lymph node enlargement in patients with PTC concomitant with HT has been unclear due to the influence of the physician’s subjective awareness of the ultrasound examination, the diversity of the nature of the nodes themselves, and the inconsistency of their description (16). Therefore, during the preoperative evaluation, the potential risks associated with LNM and HVLNM may be underestimated, potentially leading to suboptimal management of at-risk patients.

Accumulating studies have investigated in PTC concomitant with TC, particularly concerning central lymph node involvement (17–19). Additionally, some research has concentrated on identifying predictive risk factors for central lymph node metastasis (20, 21). These studies predominantly analyzed multifocal lesions and postoperative diagnoses. However, the correlation between preoperative ultrasound characteristics and HVLNM remains underexplored. Clinical decision-making could be significantly influenced by the establishment of feasible preoperative prediction models, potentially identifying the appropriateness of more aggressive treatment modalities for certain patients with PTC concomitant with HT. Therefore, the purpose of this study is to examine the factors associated with the presence of HVLNM in patients with PTC concomitant with HT, and then a predictive model of the presence of HVLNM was constructed before surgery, which is intended to assist surgeons in providing theoretical research on lymph node dissection and the extent of dissection before surgery.

## 2 Methods

### 2.1 Patients

**Figure 1:**
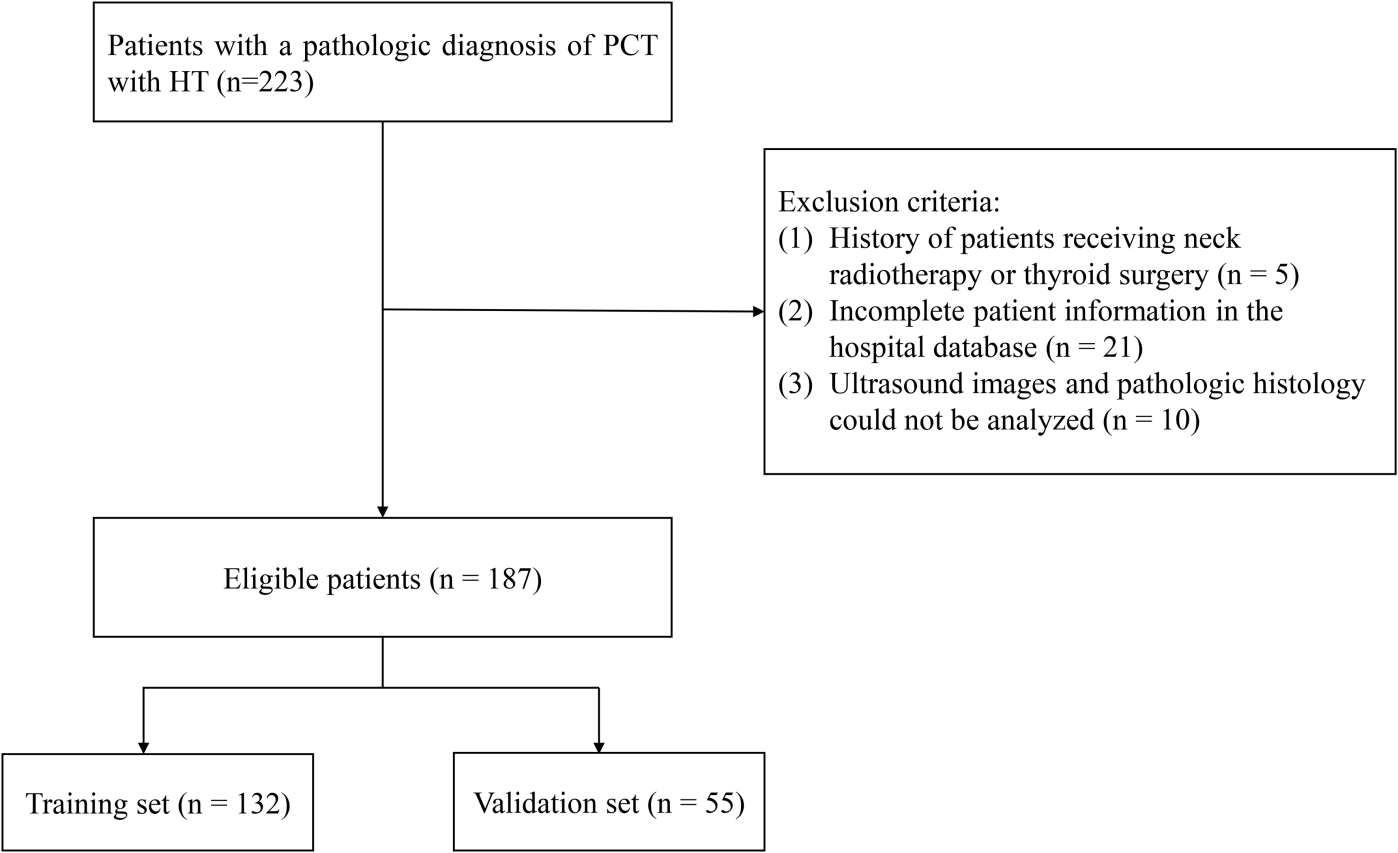
Flowchart of the study population.

In this retrospective cohort study, 223 PTC patients with Hashimoto’s thyroiditis were recruited the Affiliated Kunshan Hospital of Jiangsu University from March 2018 to December 2022. After further screening, 5 individuals were excluded from the analysis because they had undergone neck radiotherapy or thyroid surgery; 21 individuals were excluded from the analysis because their personal information in the hospital database was incomplete; and 10 individuals were excluded because their ultrasound images could not be analyzed; ultimately, a total of 187 eligible patients were included in the final analysis (**Figure 1**). The medical records and collected data, including gender, age, pathological diagnosis, blood biochemical parameters, thyroid function examination and preoperative ultrasound features were reviewed in our study. The study design has followed the Declaration of Helsinki’s guidelines. We obtained the approval of the Ethical Committee of the Affiliated Kunshan Hospital of Jiangsu University (2021-03-022-K01) for our research, and written informed consent was obtained from participants.

### 2.2 Patient selection criteria

Inclusion criteria: (1) PTC patients with postoperative pathological diagnosis of Hashimoto’s thyroiditis; (2) Adults 18 years and older; (3) Completed thyroid ultrasonography. The following criteria apply to exclusions: (1) The history of neck radiotherapy or thyroid surgery; (2) Incomplete patient information; (3) Insufficient ultrasonic image.

### 2.3 Indicators collected in the study

All patients to undergo prophylactic central lymph node dissection (CLND), as well as lateral neck lymph node dissection (LLND) if metastatic lymph nodes were suspected. Lymph node metastasis is best assessed with postoperative pathology. Postoperative pathology of more than five metastatic lymph nodes is considered high-volume lymph node metastasis (HVLNM).

Preoperative ultrasonographic analysis was performed by one experienced sonographer independently evaluating the ultrasonographic manifestations of the nodules. When the difference occurs, the image is further examined by a senior ultrasound physician. The nodules were classified according to the Chinese Thyroid Imaging Reporting and Data System (C-TIRADS) for 2020 (22). Ultrasound features were further classified as follows: tumor location (left lobe, right lobe, isthmus and both lobes), tumor nature (solid or non-solid), tumor size (≥10mm or <10mm), longitudinal to transverse ratio (≥1 or <1), tumor shape (irregular or regular), extrathyroidal extension (yes or no), histological grade (4a, 4b, 4c, 5), microcalcification (yes or no), coarse calcification (yes or no), vascularity (yes or no), multiple (≥2 or <2). Microcalcification is a hyperechoic lesion less than 1 mm. Coarse calcification is a hyperechoic lesion more than 2 mm. In ultrasound examination, thyroid capsule rupture and obvious invasion of thyroid capsule and surrounding structures were defined as extrathyroidal extension (ETE), contact and invasion were judged according to the minimum diameter of the tumor from the capsule. The presence of vascularity is defined as the detection of blood flow signals during color Doppler ultrasonography. Lymph node metastasis was examined according to postoperative pathology. Histopathological data were analyzed by senior pathologists with more than 5 years of work experience. The surgical frozen sections were used to analyze the histopathological results.

### 2.4 Statistics

In the study, patient data were entered using Excel software, and statistical analysis was performed using R software (version: 4.3.2, http://www.R-project.org). In a normal distribution, measurement data are expressed as mean ± standard deviation (SD) and compared between the two groups using Student’s t-test. Non-normally distributed measurement data are represented by a median and an interquartile range (IQR), and the Mann-Whitney U test will be used. In contrast, enumeration data are depicted as frequencies and percentages (%) and were compared using the χ2-test or Fisher’s exact test.

In linear regression models, the least absolute shrinkage and selection operator (LASSO) regression analysis selects variables and shrinks the model. A LASSO regression analysis minimizes prediction error by constraining model parameters so that some regression coefficients may shrink towards zero as a result of a constraint on the model parameters. Those variables with zero regression coefficients after shrinkage are excluded from the model, whereas those with nonzero regression coefficients are most strongly correlated. By using the type measure of -2log-likelihood and binomial family, LASSO regression in R software performs 10 times K cross-validation to centralize and normalize the included variables, then picks the optimal lambda value based on the results.”Lambda.lse” produces a model with good performance but the least number of independent variables. therefore, the LASSO method was used to further analyze the variables with *P* < 0.05 in the univariate analysis of the training set to select the optimal predictors among the current risk factors, including age, tumor nature, tumor size, longitudinal to transverse ratio, extrathyroidal extension, histological grade, microcalcification, coarse calcification, vascularity, multiple, Na, FPG and TPOAb. By introducing the feature selected in the LASSO regression model, multivariable logistic regression analysis was used to build a prediction model (23).

A predictive model is constructed based on the feature variables selected through LASSO regression analysis, including tumor size, extrathyroidal extension, histological grade, and vascularity. Collinearity analysis is used to determine whether variables are multicollinear. Constructing a nomogram model using binary logistic regression in R software. In order to evaluate the model accuracy, the receiver operating characteristic curve (ROC) was used for training set nomogram discrimination. Calibration curves and Hosmer–Lemeshow (H-L) tests were applied to determine the goodness of fit between the predicted and observed data. Internal validation of the nomogram model was conducted with bootstrap repeated sampling (1000 bootstrap resamplings). The set data were substituted into the model to predict the HVLNM. Additionally, calibration curves were plotted to verify the accuracy and consistency, and the predictive value of the prediction model was evaluated.

## 3 Results

### 3.1 Description of grouping

A total of 187 patients were randomized into 132 participants for training and 55 participants for external validation, which corresponded to the theoretical 3:1 ratio in the study. 16 (12.1%) and 6 (10.9%) patients had > 5 lymph node metastases of papillary thyroid carcinoma (PTC) with Hashimoto’s thyroiditis in the training and validation cohorts, respectively.

### 3.2 Establishment of the prediction mode

#### 3.2.1 Univariate analysis

Based on the univariate analysis, variables that were significantly associated with > 5 lymph node metastases included age (*P* < 0.010), tumor nature (*P* = 0.041), tumor size (*P* < 0.001), longitudinal to transverse ratio (*P* = 0.005), extrathyroidal extension (*P* < 0.001), histological grade (*P* < 0.001), microcalcification (*P* = 0.002), coarse calcification (*P* = 0.019), vascularity (*P* < 0.001), multiple (*P* = 0.003), Na (*P* = 0.024), FPG (*P* = 0.011) and TPOAb (*P* = 0.009) (**Table 1**). The remaining factors were not significant for identifying HVLNM (all *P* > 0.05).

**Table 1:** Comparison of features between **HVLNM (≤5)** and **HVLNM (>5)** in training set.

Factors	HVLNM		*P-*value
	≤5 (n=116)	>5 (n=16)	
Demographic parameters			
Age (years, mean (SD))	42.18 (11.19)	34.62 (6.77)	**0.010**
Gender (%)			0.525^a^
Woman	101 (87.1)	13 (81.2)	
Man	15 (12.9)	3 (18.8)	
Ultrasound features			
Tumor location (%)			0.092^a^
Left lobe	35 (30.2)	10 (62.5)	
Right lobe	65 (56.0)	5 (31.2)	
Isthmus	7 (6.0)	0 (0.0)	
Both lobes	9 (7.8)	1 (6.2)	
Tumor nature (%)			**0.041** ^a^
Solid	91 (78.4)	16 (100.0)	
Non-solid	25 (21.6)	0 (0.0)	
Tumor size (%)			**<0.001** ^a^
≥10mm	33 (28.4)	14 (87.5)	
<10mm	83 (71.6)	2 (12.5)	
Longitudinal to transverse ratio (%)			**0.005** ^a^
≥1	67 (57.8)	15 (93.7)	
<1	49 (42.2)	1 (6.3)	
Tumor shape (%)			0.126^a^
Irregular	98 (84.5)	16 (100.0)	
Regular	18 (15.5)	0 (0.0)	
Extrathyroidal extension (%)			**<0.001** ^a^
Yes	27 (23.3)	13 (81.2)	
No	89 (76.7)	3 (18.8)	
Histological grade (%)			**<0.001** ^a^
4a	32 (27.6)	1 (6.2)	
4b	52 (44.8)	5 (31.2)	
4c	30 (25.9)	6 (37.5)	
5	2 (1.7)	4 (25.0)	
Microcalcification (%)			**0.002** ^a^
Yes	62 (53.4)	15 (93.8)	
No	54 (46.6)	1 (6.2)	
Coarse calcification (%)			**0.019**
Yes	12 (10.3)	5 (31.2)	
No	104 (89.7)	11 (68.8)	
Vascularity (%)			**<0.001** ^a^
Yes	42 (36.2)	14 (87.5)	
No	74 (63.8)	2 (12.5)	
Multiple (%)			**0.003**
≥2	12 (10.3)	6 (37.5)	
<2	100 (89.7)	10 (62.5)	
Clinical hematological and biochemical parameters			
K (mmol/L, mean (SD))	4.11 (0.31)	4.11 (0.28)	0.971
Mg (mmol/L, mean (SD))	0.94 (0.07)	0.93 (0.07)	0.572
Na (mmol/L, mean (SD))	140.80 (1.87)	139.70 (1.25)	**0.024**
P (mmol/L, mean (SD))	1.14 (0.15)	1.10 (0.15)	0.431
Ca (mmol/L, mean (SD))	2.35 (0.11)	2.34 (0.08)	0.569
PT (s, mean (SD))	10.86 (0.63)	11.07 (0.63)	0.208
APTT (s, mean (SD))	27.86 (2.80)	28.30 (1.83)	0.546
INR (mean (SD))	0.92 (0.06)	0.94 (0.06)	0.233
FIB (g/L, mean (SD))	2.76 (0.54)	2.58 (0.61)	0.243
D-Dimer (mg/L, median [IQR])	0.23 [0.18, 0.35]	0.17 [0.15, 0.26]	0.067^b^
Plt (×10^9^/L, mean (SD))	249.78 (71.35)	255.96 (67.13)	0.744
Hgb (g/L, mean (SD))	127.41 (16.98)	135.41 (18.04)	0.082
ANC (×10^9^/L, median [IQR])	3.57 [2.86, 4.32]	3.75 [2.77, 4.32]	0.958^b^
ALC (×10^9^/L, mean (SD))	1.78 (0.49)	1.83 (0.51)	0.694
AMC (×10^9^/L, median [IQR])	0.33 [0.30, 0.48]	0.40 [0.30, 0.60]	0.545^b^
ALT (U/L, median [IQR])	15.00 [12.00, 23.00]	13.50 [11.50, 17.25]	0.216^b^
AST (U/L, median [IQR])	18.00 [16.00, 23.00]	17.00 [15.00, 18.50]	0.118^b^
Cr (μmol/L, median [IQR])	55.00 [47.75, 100.00]	51.00 [45.50, 84.25]	0.254^b^
BUN (mmol/L, mean (SD))	4.88 (1.31)	5.57 (1.36)	0.051
UA (μmol/L, mean (SD))	292.05 (77.36)	296.38 (65.66)	0.832
FPG (mmol/L, median [IQR])	5.12 [4.80, 5.42]	4.83 [4.58, 5.06]	**0.011** ^b^
FT4 (pmol/L, median [IQR])	13.05 [12.23, 14.63]	13.31 [12.38, 15.66]	0.378^b^
FT3 (pg/mL,median [IQR])	4.18 [3.81, 4.64]	4.44 [3.97, 4.57]	0.445^b^
T3 (nmol/L, median [IQR])	3.79 [1.61, 4.39]	3.94 [2.58, 4.52]	0.517^b^
T4 (nmol/L, median [IQR])	101.38 [84.68, 115.84]	97.14 [87.74, 114.21]	0.922^b^
TPOAb (IU/mL, median [IQR])	22.57 [3.01, 342.86]	2.13 [0.53, 8.31]	**0.009** ^b^
TGAb (IU/mL, median [IQR])	66.38 [15.60, 176.78]	83.41 [15.56, 417.06]	0.510^b^

a Fisher’s exact test;

b Mann-Whitney test;

PT, Prothrombin time; APTT, Partial thromboplastin time; INR, International normalized ratio; FIB, Fibrinogen; Plt, Platelet; Hgb, Hemoglobin; ANC, Absolute neutrophil count; ALC, Absolute lymphocyte count; AMC, Absolute monocyte count; ALT, Alanine aminotransferase; AST, Aspartate aminotransferase; Cr, Creatinine; BUN, Blood urea nitrogen; UA, Uric acid; FPG, Fasting plasma glucose; FT4, Free thyroxine; FT3, Free triiodothyronine; T3, Triiodothyronine; T4, Thyroxine; TPOAb, Thyroid peroxidase antibodies; TGAb, Thyroglobulin antibodies.The bold values represent that *P*-value was less than 0.05.

#### 3.2.2 Lasso regression analysis

The variables with significant statistical difference in the univariate analysis, were selected using the Lasso binary logistic regression model (**Supplementary Figure S1**). The tuning parameter (λ) selection in the Lasso model is based on tenfold cross-validation via the minimum criteria. The area under the binomial deviance curve was plotted versus log (λ). Dotted vertical lines were drawn at the optimal values using the minimum criteria and the 1 standard error of the minimum criteria (the 1-SE criteria). Further, log (λ) = 0.0578 was chosen (1-SE criteria) according to tenfold cross-validation of the Lasso coefficient profiles of the 13 features. A coefficient profile plot was produced against the log (λ) sequence. A vertical line was drawn at the value selected using tenfold cross-validation, where optimal λ resulted in 4 nonzero coefficients. They were tumor size, extrathyroidal extension, histological grade and vascularity.

#### 3.2.3 Collinearity diagnostic analysis

To further quantify the severity of collinearity, we used the tolerance and VIF based on the Lasso regression analysis. Results indicated that each variable’s tolerances were greater than 0.2, and the VIFs were all greater than 5, suggesting no significant cross-collinearity (**Supplementary Table S1**).

#### 3.2.4 Multivariate analysis

We included four variables in the binary logistic regression that did not have any collinearity (**Supplementary Table S2**). In the binary logistic regression analysis, tumor size (*P* = 0.023), extrathyroidal extension (*P*  = 0.023), histological grade (4b: *P* = 0.457, 4c: *P* = 0.303, 5: *P* =  0.052) and vascularity (*P* =  0.098) are shown in **Table 2**. To predict HVLNM, a logistic regression model was constructed based on the logistic regression coefficient and the constant term. The logistic regression equation was as follows: Logit (P) = -6.221 + 1.979 × Tumor size + 1.759 × Extrathyroidal extension + 0.915× Histological grade (4b) + 1.283 × Histological grade (4c) + 3.497 × Histological grade (5) + 1.524 × Vascularity.

**Table 2:** Coefficients of binary logistic regression for predicting HVLNM (>5) in training set.

Variables	β	S.E.	Wald	*P-*value	OR	95% CI for OR
Tumor size						
<10mm	−	−	−	−	Reference	−
≥10mm	1.979	0.868	2.280	0.023	7.232	1.540-54.582
Extrathyroidal extension						
No	−	−	−	−	Reference	−
Yes	1.759	0.771	2.282	0.023	5.807	1.382-30.974
Histological grade						
4a	−	−	−	−	Reference	−
4b	0.915	1.229	0.745	0.457	2.497	0.289-55.507
4c	1.283	1.245	1.031	0.303	3.606	0.402-81.472
5	3.497	1.797	1.946	0.052	33.011	1.410-1853.152
Vascularity						
No	−	−	−	−	Reference	−
Yes	1.524	0.920	1.657	0.098	4.589	0.858-36.657
Constant	-6.221	1.497	-4.155	<0.001	−	−

S.E., Standard error; OR, Odds ratio; CI, Confidence interval.

Logit (P) = -6.221 + 1.979 × Tumor size + 1.759 × Extrathyroidal extension + 0.915× Histological grade (4b) + 1.283 × Histological grade (4c) + 3.497 × Histological grade (5) + 1.524 × Vascularity.

Hosmer-Lemeshow Goodness-of-fit test:P = 0.297.

#### 3.2.5 Establishment of the nomogram model

**Figure 2:**
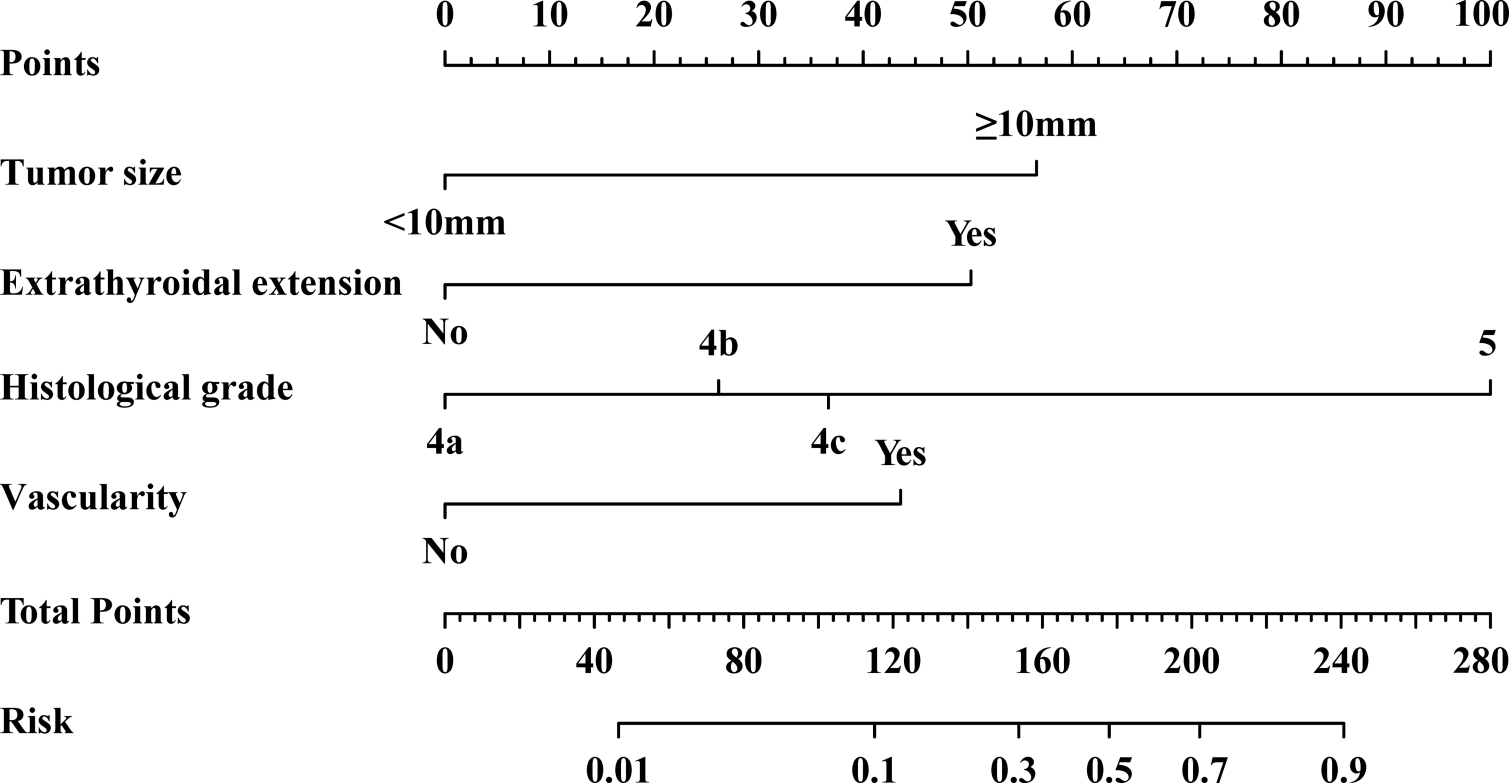
Nomogram for predicting the HVLNM (>5) in the training set.

According to the results of binary logistic regression analysis, the nomogram model was constructed using R software (**Figure 2**). Based on the measurements of tumor size, extrathyroidal extension, histological grade, and vascularity in each patient, a line perpendicular to the axis of the corresponding indicator was drawn on the nomogram, and the points of each indicator were added. A line perpendicular to the risk axis, which represents the probability of HVLNM, was drawn following the sum on the total points line.

#### 3.2.6 Evaluation of the nomogram model in the training set

**Figure 3:**
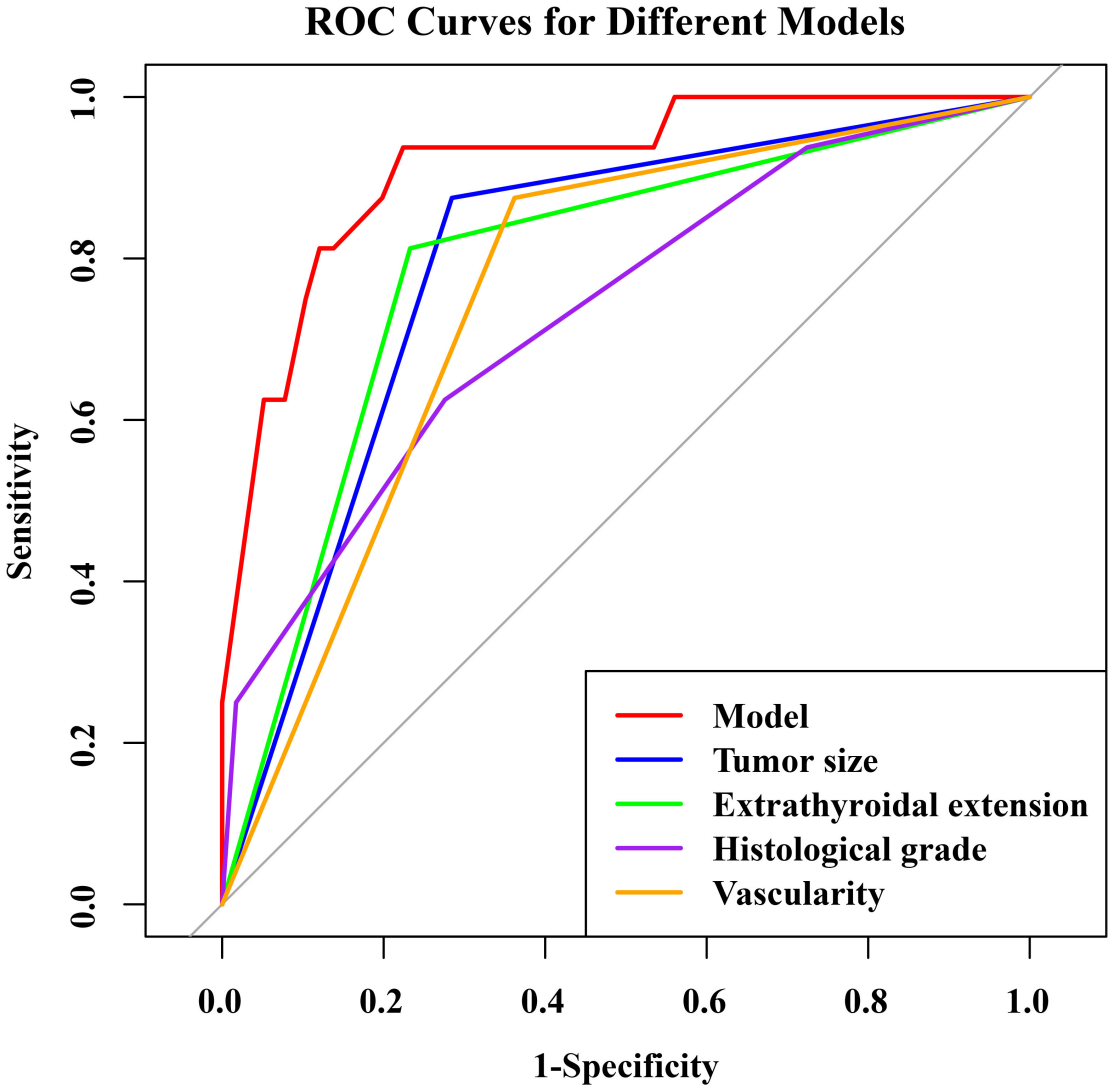
The ROC curve of the combined predictive model for predicting HVLNM (>5) in training set.

**Figure 4:**
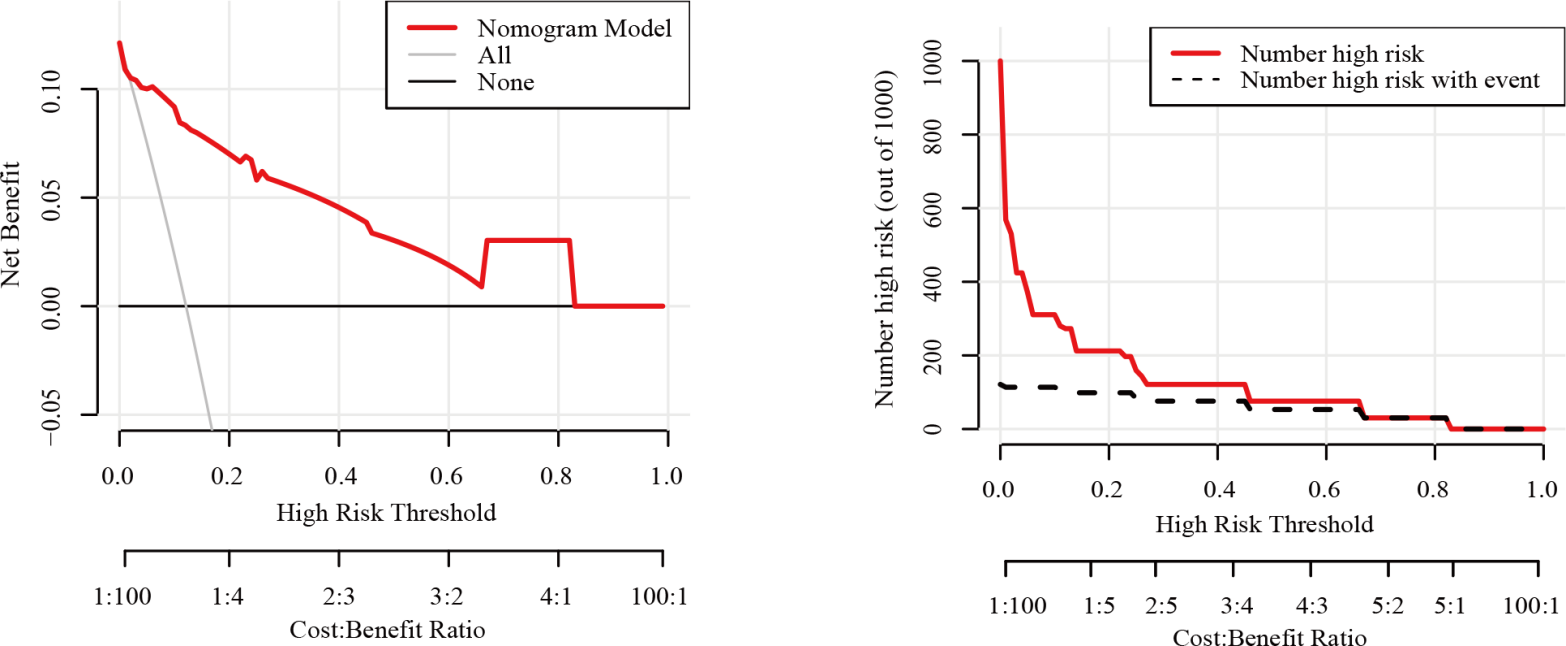
Decision curve analysis for the nomogram model in the training set..

The nomogram had an AUC of 0.914 (95% CI: 0.841–0.987), sensitivity of 93.8%, specificity of 77.6% and accuracy of 79.5% (**Table 3**, **Figure 3**). The AUCs of tumor size, extrathyroidal extension, histological grade and vascularity were 0.795 (95% CI: 0.702–0.889), 0.790 (95% CI: 0.684–0.896), 0.734 (95% CI: 0.602-0.864) and 0.757 (95% CI: 0.662–0.851). There was a p-value of 0.297 for the Hosmer–Lemeshow-Goodness-of-Fit test, and the calibration plot is shown in **Supplementary Figure S2**. As shown in **Figure 4**, the decision curve analysis (DCA) showed good net benefits.

**Table 3:** The ROC analysis of tumor size, extrathyroidal extension, histological grade, vascularity and the model in the training set.

	AUC (95% CI)	ACC (%)	SN (%)	SP (%)	*P*-value
Tumor size	0.795 (0.702-0.889)	73.5	87.5	71.5	<0.001
Extrathyroidal extension	0.790 (0.684-0.896)	77.3	81.3	76.7	<0.001
Histological grade	0.734 (0.602-0.864)	71.2	62.5	72.4	<0.001
Vascularity	0.757 (0.662-0.851)	66.7	87.5	63.8	<0.001
Model	0.914 (0.841-0.987)	79.5	93.8	77.6	<0.001

AUC, Area under curve; ACC, Accuracy; SN, Sensitivity; SP, Specificity; *P*-value, DeLong test of AUC; CI, Confidence interval.

### 3.3 Internal validation of the nomogram model

**Figure 5:**
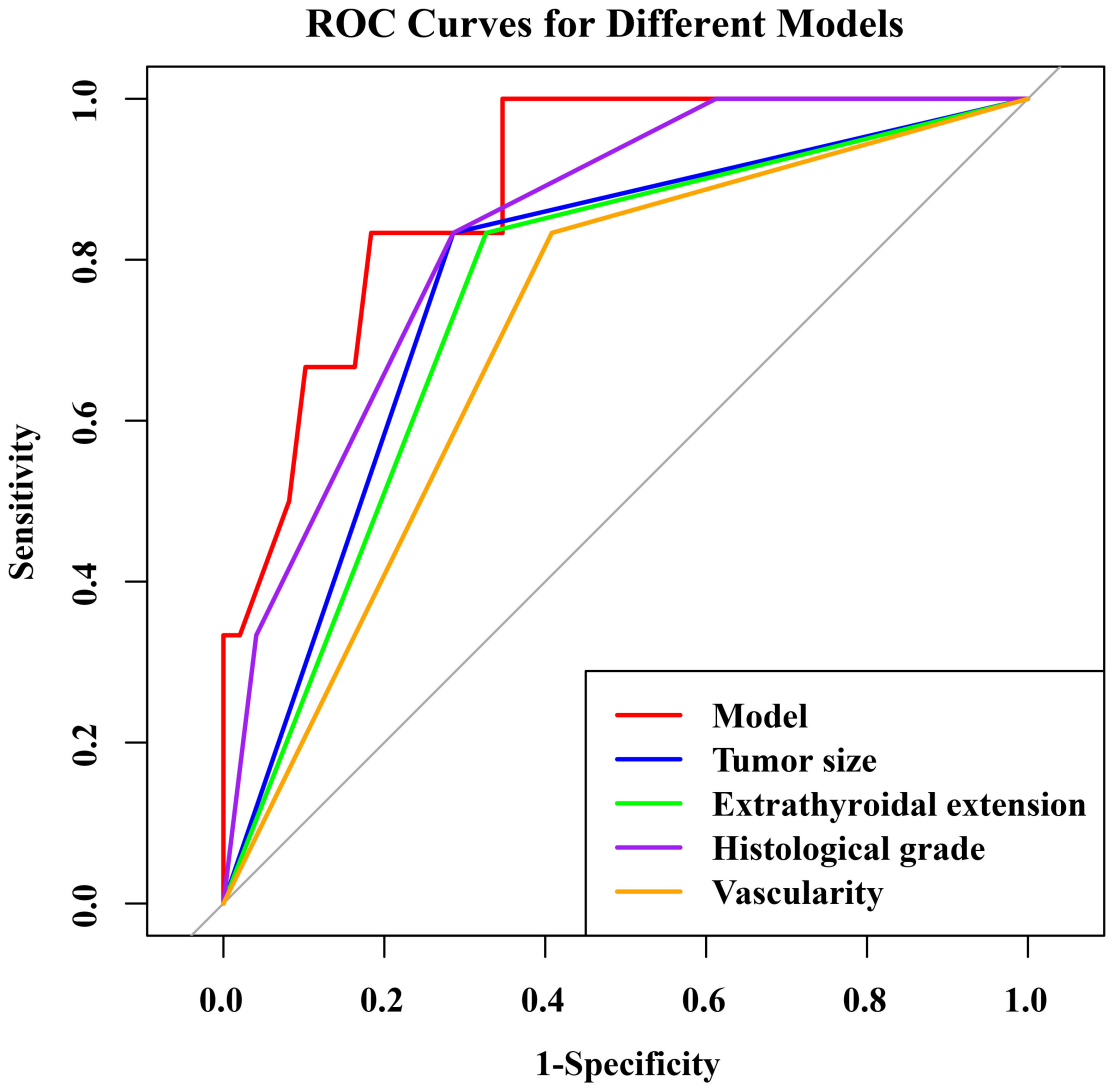
The ROC curve of the combined predictive model for predicting HVLNM (>5) in testing set.

**Figure 6:**
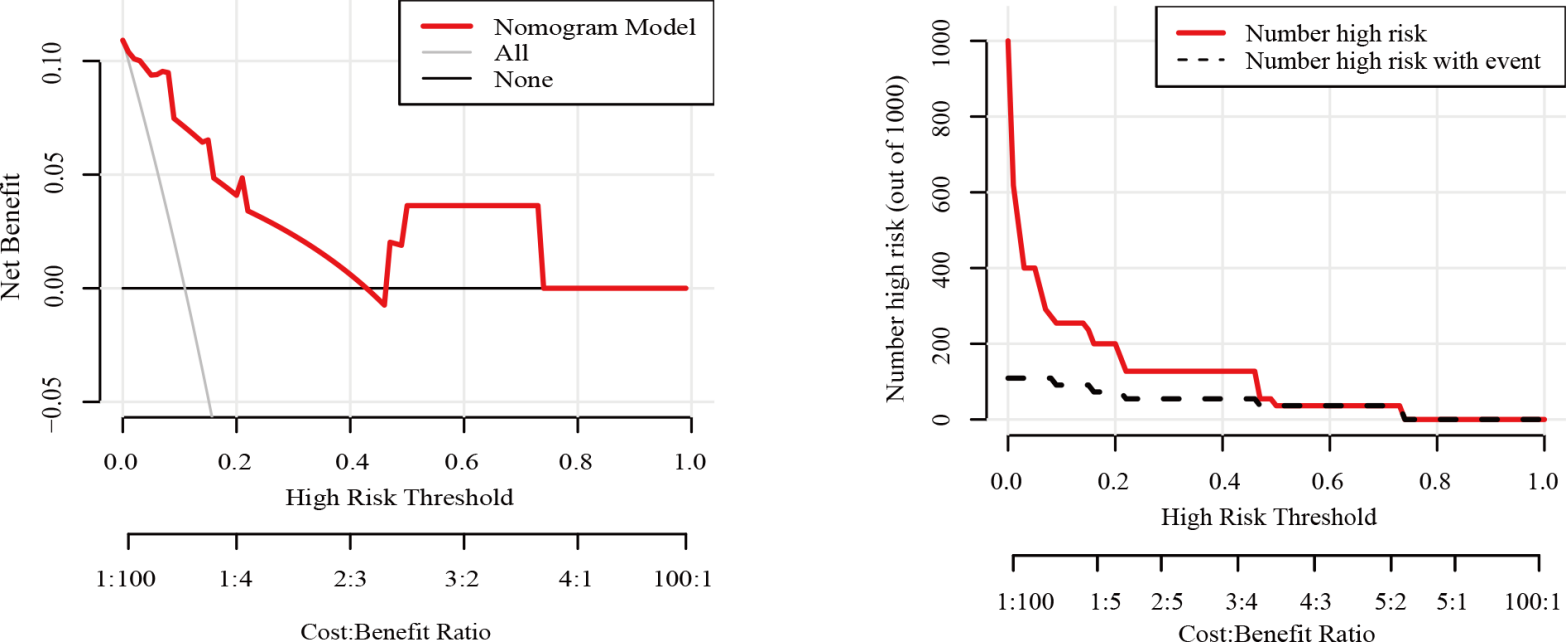
Decision curve analysis for the nomogram model in the testing set.

To ensure that the model did not overfit the training data, the bootstrap repeated sampling method was used for internal validation in this study. The accuracy was 87.3%, suggesting that the model did not overfit (**Table 4**). The AUC of the prediction model for the validation group was 0.889 (**Supplementary Table S3**, **Figure 5**). There was excellent agreement between the nomogram predictions and the actual values according to the internal calibration plots (**Supplementary Figure S3**). The DCA had good net benefits in the validation group (**Figure 6**).

**Table 4:** Predictive values of nomogram model in internal validation set.

Nomogram model-predicted efficacy outcome	Clinical standard-based follow-up outcome		Total
	HVLNM (>5)	HVLNM (≤5)	
HVLNM (>5)	3	4	7
HVLNM (≤5)	3	45	48
Total	6	49	55

The accuracy of the model in the validation set were 87.3%.

## 4 Discussion

Recently, several studies have found an increase in thyroid cancer cases associated with Hashimoto’s thyroiditis (HT) (24), and the incidence of PTC coexists with HT is more frequent. Evidence suggests that the prognosis of PTC patients is related to the number and size of the involved lymph nodes. Research from Randolph et al. have demonstrated that in patients with pathological staging of N1, there is a significant difference in tumor recurrence risk between those with fewer than 5 positive lymph nodes and those with more than 5 positive lymph nodes (10). The National Cancer Data Base and the SEER database revealed a lower overall survival rate with more metastatic lymph nodes (up to six metastatic). Conversely, more positive nodes were not associated with an increased mortality risk (11). As a result, the 2015 ATA guidelines have incorporated clinical and pathological nodal status into stratifying the PTC recurrence risk. A lymph node with more than five metastatic lymph nodes is considered intermediate risk, indicating a greater than 20% possibility of recurrence (9). Predicting large-volume lymph node metastasis, distinguishing between benign and malignant lymph nodes, and determining the extent of lymph node dissection are essential for PTC patients with HT. Hence, we developed a nomogram based on ultrasound and clinicopathological characteristics of lymph nodes that predicts the occurrence of large-volume lymph node metastases in this study.

In the current study, tumor size, extrathyroidal extension, histological grade, and vascularity were independent predictors of large-volume lymph node metastasis. Based on these parameters, a nomogram to predict large-volume lymph node metastasis was constructed, and our results showed that its AUC was 0.914. The histological grade is more important in the built nomogram. Histologic grading has been found to have an impact on the prognosis of papillary thyroid cancer, with findings showing that worsening histologic grading of papillary thyroid cancer was independently associated with a parallel increase in the risk of death that approached or exceeded identified risk factors for thyroid cancer (25). Our finding aligns with several studies that have explored similar predictive models and the associated risk factors for lymph node metastasis in PTC patients. A study developed a nomogram for predicting central lymph node metastasis (CLNM) in PTC patients with HT, and they found that tumor size, age, and ultrasound characteristics were significant predictors. This study emphasized the utility of a nomogram to guide clinical decisions and improve patient management, which was similar to our findings on the importance of tumor size and histological feature (26). Another relevant study focused on papillary thyroid microcarcinoma (PTMC), and this study constructed a nomogram incorporating tumor size, ultrasound features, and patient age to predict high-volume central lymph node metastasis (HVLNM), which found that patients with larger tumor sizes and certain ultrasound characteristics had a higher risk of HVLNM, corroborating the significance of tumor size and extrathyroidal extension noted in our research (12).

Tumor size is an independent predictor of large-volume LNM in our research. Researchers found that papillary thyroid microcarcinoma (PTMC) patients with larger tumors were more likely to develop high-risk LNM (27). Similarly, another study found that tumor diameter was an important predictor of LNM, emphasizing its importance for treatment planning and prognosis (28). According to other research, extensive nodal metastases are associated with extrathyroidal extension (ETE). In T1 papillary thyroid cancer, extranodal extension was independently associated with extensive nodal metastasis (28). Our research also considered the histological grade as a predictor. While not all studies focus on histological grading, some do acknowledge its role. PTMC risk could be predicted using a nomogram incorporating histological factors, along with other variables, indicating the relevance of histopathology in predicting metastasis risk (29).

Zhao et al. retrospectively collected data from 994 patients with PTC combined with HT, of which 606 patients with relatively early surgery were used as a training cohort for building the column line graphs, and 388 patients with relatively late surgery were used as a validation cohort for verifying the performance of the model. The results of the study showed that younger age, normal body mass index, BRAFV600E mutation, larger maximum diameter, left lobe tumor, aspect ratio >1, envelope invasion and calcification were significant risk factors for central lymph node metastasis in patients with PTC combined with HT (19). The findings of the results of this study were significantly different from those of our study, and the possible reason for this may be that the sample size of the present study was too small, in addition to the fact that some of the information was not fully available, and therefore the predictors derived were different from those of the previous studies. In addition, Zhu et al. devised and validated a clinical predictive model for HVLNM of PTC. They found that young age is an independent risk factor for HVLNM in PTC patients. Also, their study identified a complex nonlinear association between age and HVLNM that may affect the diagnostic efficacy of the model in specific age subgroups (30). Considering the study population and the sample size, we did not find age to be a predictor in our study, and further studies are needed to confirm this point.

Currently, total thyroidectomy is the treatment of choice for benign multinodular goiter. Up to one-third of patients undergoing total or complete thyroidectomy develop postoperative hypocalcemia, which is the most common complication resulting from parathyroid insufficiency (31). In addition, hemithyroidectomy should always be used as the initial procedure for patients with thyroid nodular disease with indeterminate cytologic results. This surgical strategy avoids unnecessary overtreatment and the complications and overall costs associated with total thyroidectomy, and does not increase the risk of complications if total thyroidectomy is required (32). Therefore, to some extent, the results of this study may give clinicians options to avoid unnecessary total thyroidectomy, which can better minimize patient complications.

There are still some limitations to our research. First, the data in this study came from the same hospital, which is relatively homogeneous and may have a case selection bias; second, the sample size of this study is small, so it needs to be combined with multicenter large-sample data analysis and evaluation to improve the efficacy of the model. Finally, this study is retrospective, and prospective studies are needed to elucidate the relationship between papillary thyroid cancer and Hashimoto’s thyroiditis.

## 5 Conclusion

In conclusion, our study establishes a nomogram integrating papillary thyroid carcinoma lesions, ultrasound features and clinicopathologic features to predict high volume lymph node metastasis in PTC Hashimoto’s thyroiditis. Tumor size, extrathyroidal extension, histological grading, and vascularity are important predictors of high-volume lymph node metastasis, and these parameters are expected to provide useful information to guide patients to the appropriate intraoperative window and develop a personalized treatment plan for these PTC patients.

## Data Availability

The original contributions presented in the study are included in the article/**Supplementary Material**. Further inquiries can be directed to the corresponding author.
