# Supplementary material for: A multivariable model of ultrasound and biochemical parameters for predicting high-volume lymph node metastases of papillary thyroid carcinoma with Hashimoto’s thyroiditis
Source: Front Endocrinol (Lausanne). 2025 Jan 10;15:1501142. doi: 10.3389/fendo.2024.1501142 (PMC11757122; doi:10.3389/fendo.2024.1501142)
Supplement: Supplementary file 1 [file DataSheet1.docx]

Supplementary Material


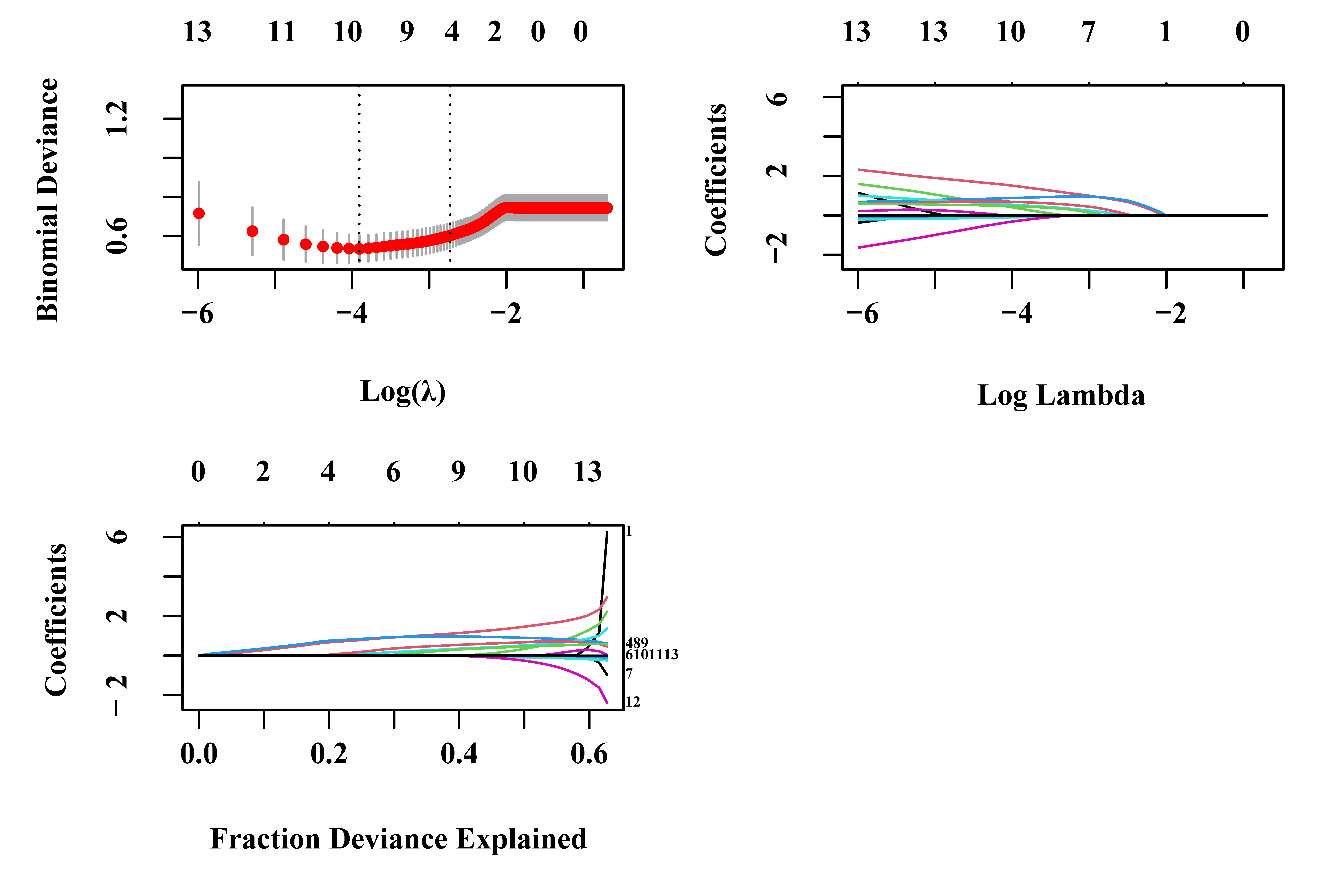


Figure S1. The Coefficients of LASSO regression analysis.


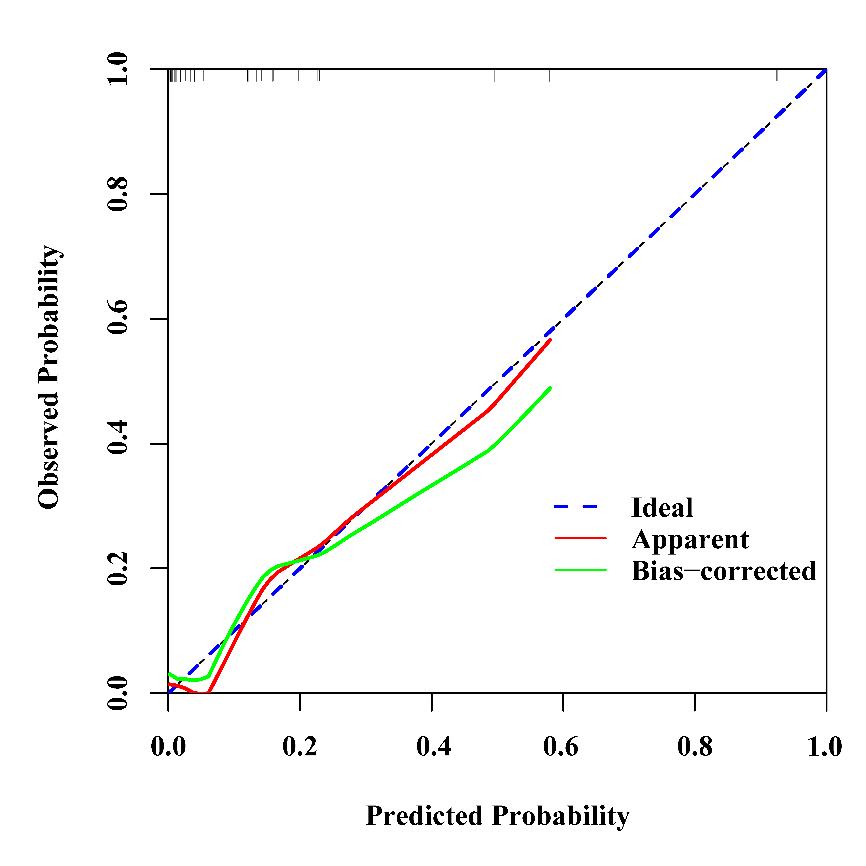


Figure S2. Calibration plot of the model in the training set.


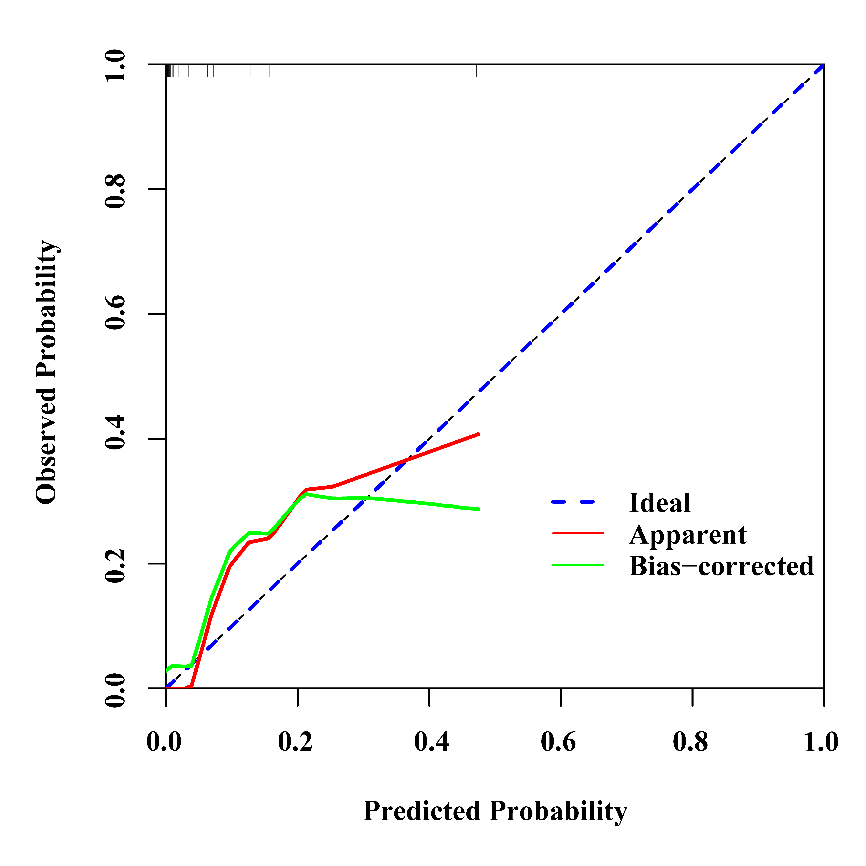


Figure S3. Calibration plot of the model in the testing set.

Table S1. The collinearity diagnostic analysis of factors for predicting HVLNM (>5) in training set.

| Variables | Tumor size | Extrathyroidal extension | Histological grade | Vascularity |
| --- | --- | --- | --- | --- |
| Tolerance | 0.809 | 0.808 | 0.900 | 0.813 |
| VIF | 1.236 | 1.238 | 1.111 | 1.230 |

Table S2. The Spearman's rank correlation coefficient of factors for predicting HVLNM (>5) in training set.

|  | Tumor size | Extrathyroidal extension | Histological grade | Vascularity |
| --- | --- | --- | --- | --- |
| Tumor size | 1.000 | 0.301 | 0.156 | 0.386 |
| Extrathyroidal extension | 0.301 | 1.000 | 0.302 | 0.301 |
| Histological grade | 0.156 | 0.302 | 1.000 | 0.103 |
| Vascularity | 0.386 | 0.301 | 0.103 | 1.000 |

Table S3. The ROC analysis of tumor size, extrathyroidal extension, histological grade, vascularity and the model in the testing set.

|  | AUC (95% CI) | ACC (%) | SN (%) | SP (%) | *P*-value |
| --- | --- | --- | --- | --- | --- |
| Tumor size | 0.774 (0.593-0.965) | 72.7 | 83.3 | 71.4 | 0.004 |
| Extrathyroidal extension | 0.753 (0.559-0.948) | 69.1 | 83.3 | 67.3 | 0.009 |
| Histological grade | 0.837 (0.693-0.980) | 72.7 | 83.3 | 71.4 | 0.003 |
| Vascularity | 0.713 (0.512-0.913) | 61.8 | 83.3 | 51.9 | 0.026 |
| Model | 0.890 (0.771-0.999) | 69.1 | 100% | 65.3% | <0.001 |

Abbreviations: AUC, Area under curve; ACC, Accuracy; SN, Sensitivity; SP, Specificity; *P*-value, DeLong test of AUC; CI, Confidence interval.
